# Supplementary figures and images for: Proteolytic dissection of eIF4G reveals the closed-loop mRNP as an architecture for translation repression
Source: bioRxiv. 2026 Apr 7:2026.04.06.716749. Preprint. [Version 1] doi: 10.64898/2026.04.06.716749 (PMC13081929; doi:10.64898/2026.04.06.716749)

Supplemental Figure 1

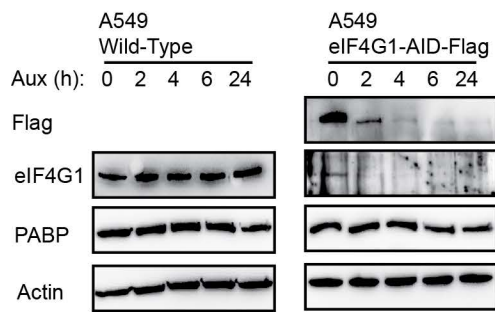

Supplemental figure 2

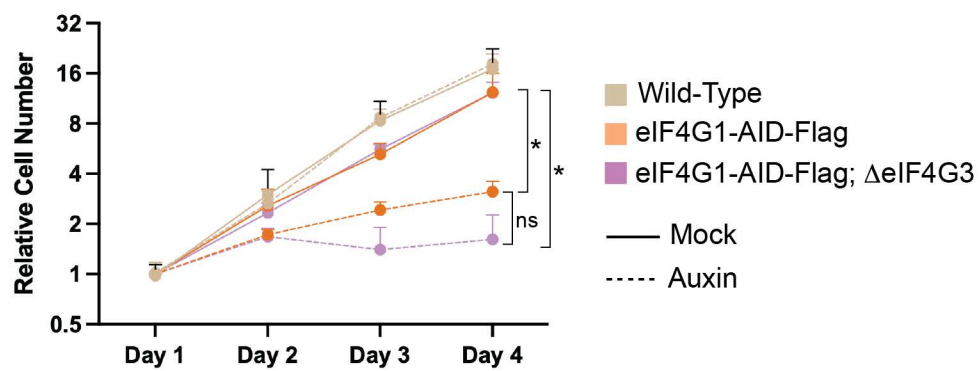

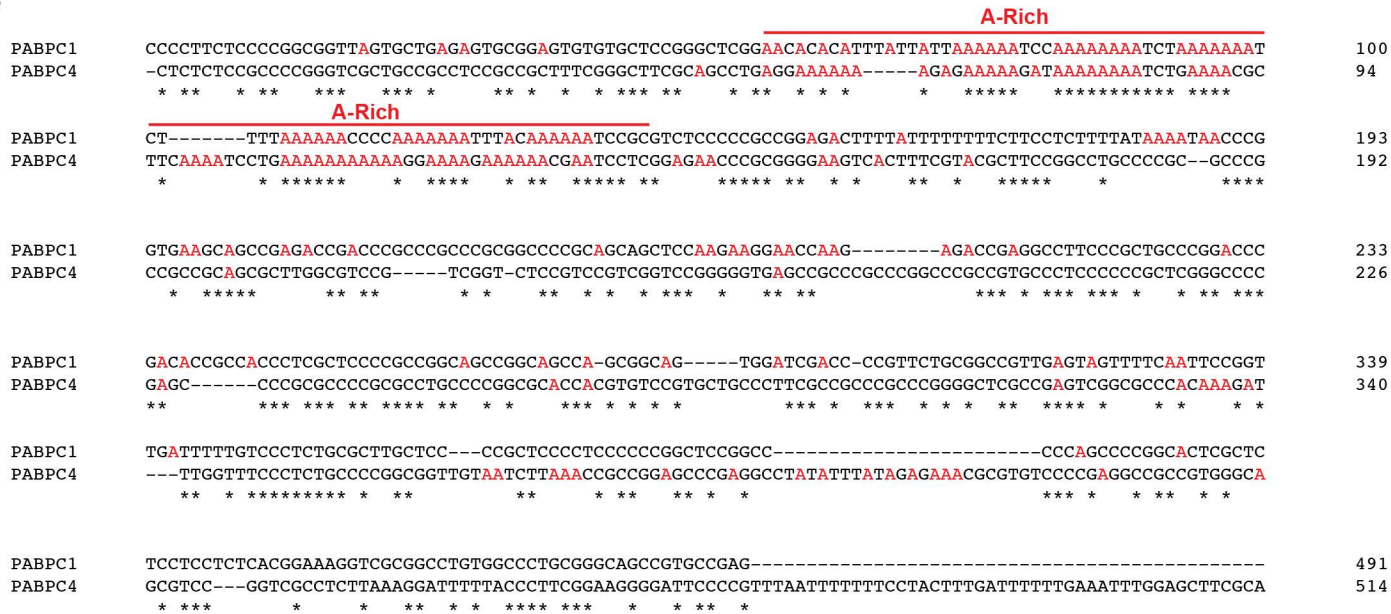

Supplement: 1 [file NIHPP2026.04.06.716749V1-supplement-1.pdf]
